# Frequency and Predictors of Alcohol-Related Outcomes Following Alcohol Residential Rehabilitation Programs: A 12-Month Follow-Up Study

**DOI:** 10.3390/ijerph16050722

**Published:** 2019-02-28

**Authors:** Elena Fiabane, Lorenza Scotti, Antonella Zambon, Giovanni Vittadini, Ines Giorgi

**Affiliations:** 1 Department of Physical and Rehabilitation Medicine, ICS Maugeri Spa SB, Institute of Genoa Nervi, via Missolungi, 14, 16167 Genoa, Italy; elenamaria.fiabane@unipv.it; 2 Department of Statistics and Quantitative Methods, University of Milano-Bicocca, Via Bicocca degli Arcimboldi, 8, 20126 Milano, Italy; antonella.zambon@unimib.it; 3 U.O. Riabilitazione Alcologica, ICS Maugeri Spa SB, IRCCS, Via S. Maugeri, 10, 27100 Pavia, Italy; vittadinigiovanni@gmail.com; 4 Servizio di Psicologia, ICS Maugeri Spa SB, IRCCS, Via S. Maugeri, 10, 27100 Pavia, Italy; ines.giorgi@icsmaugeri.it

**Keywords:** alcohol dependence, abstinence, hospitalization, residential rehabilitation, chronicity

## Abstract

Excessive use of alcohol has been identified as a major risk factor for diseases, injury conditions and increased mortality. The aims of this study were to estimate the frequency of success (abstinence and no alcohol related hospitalization) at 6- and 12-month follow-up after hospital discharge, and to identify the predictors of success. In 2009, a total of 1040 patients at their first admission in one of the 12 Residential Alcohol Abuse Rehabilitation Units (RAARUs) participating in the CORRAL (COordinamento of Residenzialità Riabilitative ALcologiche) project were included in the study. Several socio-demographic and clinical variables, and the number of treatments’ strategies during the rehabilitation were collected. Information on alcohol abstinence and no alcohol related hospitalization was assessed through a phone interview using a health worker-administered structured questionnaire at six and 12 months after discharge. An inverse probability weighted, repeated measures Poisson regression model with robust variance was applied to estimate the association between patients’ characteristics and the study’s outcomes, accounting for non-responders status. The frequencies of abstinence and non-alcohol related hospitalization were 68.38% and 90.73% at six months, respectively, and 68.65% and 87.6% at 12 months, respectively. Patients that were already abstainers in the month before RAARUs’ admission have an increased probability of being abstainers after discharge (relative risk: RR 1.20, 95% confidence interval: 95%CI 1.08–1.33) and of having an alcohol related hospitalization at 12 months. Subjects undergoing more than four treatment strategies (RR 1.19; 95% CI 1.01–1.40) had a higher abstinence probability and lower probability of no alcohol related hospitalizations after 12 months. Finally, patients with dual diagnosis (co-occurrence of alcohol abuse/dependence and psychiatric disorders) have a decreased probability of not being hospitalized for alcohol-related problems (RR 0.95; 95% CI 0.91–0.99). The results of this study suggest that specific attention should be paid to the intensity of treatment, with particular regard to a multidisciplinary rehabilitation in order to respond to the complexity of alcohol dependent patients.

## 1. Introduction

Alcohol use disorder (AUD) causes a large number of health problems and places a significant social and economic burden on societies. It is responsible for more than 200 diseases and injury conditions in individuals, including alcohol dependence, liver cirrhosis, cancers and injuries [1,2,3]. The World Health Organization reported that in 2012 about 3.3 million deaths, or 5.9% of all global deaths, were attributable to alcohol consumption [2]. In addition, AUD is also responsible for serious social and economic consequences for both drinkers and non-drinkers [3]. For these reasons, the prevention and the treatment of alcohol dependence is a public health priority and one of the challenges of the World Health Organization in order to reduce the health and social burden.

The treatment of AUD currently involves a wide variety of effective treatment options which can be performed either in an inpatient or outpatient setting, including medications (e.g., disulfiram), cognitive behavioral therapy (CBT), motivational enhancement therapy, 12-step facilita­tion therapy, contingency management, relapse prevention therapy, and family therapy [4,5,6,7].

Among treatment options, hospitalization in structures devoted to the treatment of the addictions (specifically, the Residential Alcohol Abuse Rehabilitation Units—RAARUs) is an increasingly used alternative to outpatients’ treatments. RAARUs provide a treatment pattern characterized by shortness of hospitalization (28 days); intensity of intervention (physical, psychological and family-related); complexity of intervention itself (with provision of medical, psychological and educational services); multi-professional intervention; and elaboration of an aftercare project [5]. This therapeutic approach is inspired by the American experience of the Minnesota Model [8], the Croatian psychiatry studies [9], and the German and Austrian psychosomatic clinics [10]. RAARUs are designed to detoxify the individual and to enhance their motivation to change, coping strategies, and interpersonal skills [4,5,11].

Previous studies showed high relapse rates (around 60%) within the first six months following detoxification and explored the prognostic factors for patients’ outcomes [5,12,13,14,15,16,17,18,19,20,21]. This high rate of relapse can be partially explained by the complexity and dynamicity of the addictive change process [17]. The majority of previous studies which examined predictors of posttreatment relapses in patients with alcohol dependence showed that relapse is a complex and dynamic event that appears to be determined by biological, psychological, and social factors, and an interaction among these [4,15,16]. Literature which focused on socio-demographic factors found that males of a lower age, with a low educational level, unemployment and low socio-economic level, seem to be negatively associated with alcohol-related outcomes [14,16,21,22,23]. Moreover, several studies found that clinical aspects such as dependence severity [13], amount of alcohol consumption [16], polysubstance abuse [4], and a comorbid medical condition [23] are relevant prognostic factors for relapse. With regard to treatment-related factors, previous studies found that the number of previous treatments or detoxifications [21] and treatment dropout [24] are significant predictors of relapse. Terra and colleagues [21] showed, in a sample of 300 Brazilian alcoholic patients, that previous treatment for alcohol dependence and being single were significantly associated with relapse at six months following treatment. Moreover, several studies have reported a high prevalence of co-occurrence of alcohol use disorder and psychiatric disorders [25,26,27,28,29]. However, the condition where alcohol dependence is accompanied by another mental disturbance can be underestimated because patients asking for a psychiatric consultation usually do not spontaneously reveal their risk behaviors related to drinking [30]. For example, Martinotti et al. [28] found high proportions of alcohol users in psychiatric patients, ranging from 65.5% (patients with a diagnosis of schizophrenia) to 88.9% (patients with a diagnosis of bipolar disorder). A recent study [27] examined change over the last 20 years of alcohol-dependent patients and found a significant increment of patients admitted to residential programs for alcohol dependence with polysubstance abuse and/or dual psychiatric diagnosis. However, a limited number of studies focused on the relationship between dual diagnosis and treatment outcomes [19], and many studies exploring predictors of posttreatment drinking outcomes included relatively small sample sizes [14] enrolled from one clinic [15], which reduces the generalizability of results.

Since few studies have provided the frequency of alcohol-related outcomes after inpatient alcohol rehabilitation, and studies that aimed to investigate the predictors of these outcomes provided inconclusive results, the aims of this study were (a) to estimate the frequency of success (abstinence or no alcohol related hospitalization) at 6- and 12-months after hospital discharge; (b) to identify sociodemographic and clinical characteristics and intensity of treatment predictors of success among patients with alcohol dependence problems undergoing inpatient rehabilitation programs.

## 2. Materials and Methods

### 2.1. Patients

The data analyzed in this study were collected from patients recruited within the CORRAL (COordinamento of Residenzialità Riabilitative ALcologiche) project. The CORRAL project included 12 RAARUs—public or private hospitals specialized in the management of psychiatric disorders and alcohol dependence—with 10 in the north of Italy, 1 in the centre and 1 in the south. To the best of our knowledge, CORRAL is the only nationwide Italian project carried out with the aim of investigating the socio-demographic and clinical variables and treatment patterns of patients undergoing inpatient rehabilitation for alcohol abuse or dependence. A total of 1040 patients hospitalized during 2009 were included in this study. Patients with more than one admission during this year were recruited at their first hospitalization. Patients could have been sent to the RAARUs by their relatives, physician, or public outpatients’ services dedicated to the treatment, prevention and rehabilitation of addictions.

### 2.2. Assessment of Patients’ Sociodemographic and Clinical Characteristics and Treatment’s Intensity

At the beginning of the hospital stay, patients were interviewed by a trained health worker with extensive experience in the alcoholic rehabilitation field, using a structured 26-item questionnaire. In this study, as potential predictors of alcohol-related outcomes, we considered several socio-demographic (age, gender, education, occupation) and clinical characteristics (comorbidities including cirrhosis, liver disease except cirrhosis, polyneuropathy and other comorbidities), alcohol abstinence in the last month before hospital admission, polysubstance abuse, and dual diagnosis, the latter assessed by a psychiatrist according to DSM-IV-TR criteria (co-occurrence of alcohol abuse/dependence and at least one diagnosis among drugs dependence or abuse, personality disorders, affective syndrome, anxious and reactive syndrome, schizophrenic psychosis, impulse control disorder, dementia and oligophrenia). Alcohol abstinence in the last month before hospital admission was verified by medical clinical evaluation and by blood tests related to liver function parameters (i.e., serum γ-glutamyltransferase—GGT).

Finally, the treatment strategies during the hospital stay (expressive activities, formative program, psychological group program, psychological individual program, psychological symptoms therapy, psychological family program, adverse effects therapy and abstinence syndrome therapy) were identified from the medical records of the patients at discharge. The number of different treatment strategies during hospital stay was categorized in three classes as ≤2, 3–4 and >4 representing a low, medium, and high treatment intensity. Treatment intensity can be considered a proxy of the attention of the RAARUs’ members to the complexity of the patients’ condition.

### 2.3. Assessment of Outcomes during Follow-Up

Patients included in the study were contacted by telephone at 6 and 12 months after discharge by the same health worker that performed the inpatient interview. The follow-up questionnaire included questions regarding the onset of alcohol-related problems. The interviewers were trained in doing in-depth interviews about the actual nature of the reported alcohol-related problems. Specifically, the alcohol use (complete abstinence, reduction or no reduction) and the occurrence of alcohol-related hospitalizations (hospital and/or emergency room admissions) were recorded. The success of the rehabilitation process was defined by complete alcohol abstinence (AA) or no alcohol related hospitalizations (NARH). Patients who were not reachable or who refused to provide information regarding AA or NARH at 6 (205 for AA and 220 for NARH) or 12 months (265 for AA and 264 for NARH) were defined as non-responders for the specific outcome at that time point.

### 2.4. Statistical Methods

All analyses were performed separately for AA and NARH outcomes.

Categorical variables were reported as absolute and relative frequencies. Since a high proportion of non-responders was expected, the differences of potential predictors between responders and non-responders were evaluated by *Χ*^2^ test. To take into account of the potential selection bias due to non-responders, the high frequency of outcome, and to analyze the outcomes at 6 and 12 months together, we applied the inverse probability weighting approach to a repeated measures Poisson regression model (IPW-RMP) with robust variance. Briefly, in these regression models all patients were weighted by their corresponding inverse probability of being a non-responder at 6 or 12 months. The probability of being a non-responder was estimated by means of a logistic regression model including only covariates significantly associated to the responding status according to the *Χ*^2^ test.

Univariate IPW-RMP models were fitted to estimate the association between each potential predictor and the risk of each outcome and to select which variables should be included in the multivariate model. Dichotomous predictors were considered eligible for inclusion in the multivariate model if the corresponding *p*-value was ≤ 0.2. Categorical variables with more than two levels were considered eligible when the *p*-value was ≤ 0.2 for at least one level. An initial multivariate model was fitted including the variables identified as eligible at the univariate analysis (i.e., age, gender, follow-up time (6 and 12 months), the interaction terms between covariates, and time to evaluate if the covariate effects changed over time). Statistically significant interaction terms only were retained in the final model. The association estimates were reported as relative risks (RRs) with their corresponding 95% confidence intervals (95% CI).

Data were analyzed using SAS v 9.4 (SAS Institute, Cary, NC, USA); all tests were two-tailed and those with a *p*-value ≤ 0.05 were considered statistically significant.

## 3. Results

Overall, 1040 patients (mean age, 46 years; SD 11.02; proportion that were male, 71.63%) were included in the analyses. Data on AA outcome was available for 835 (80.29%) patients at 6 months and 775 (74.52%) at 12 months. The information on NARH was available for 820 (75.85%) and 776 (74.61%) patients at 6 and 12 months, respectively.

Table 1 reports the distribution of responders and non-responders of both outcomes at 6 and 12 months according to sociodemographic and clinical characteristics and the *p*-value of the *Χ*^2^ test. At 6 and 12 months, the age distribution and proportion of polysubstance abusers differed between responder and non-responder for both outcomes. In particular, younger patients (age ≤ 40 years) and polysubstance abusers tended to more frequently be non-responders. The proportion of unemployed patients differs for both outcomes at 6 months. Differences in the proportion of abstainers the month before rehabilitation period and in the proportion of patients with dual diagnosis was observed only at 12 months for both AA and NARH.

The overall proportion of AA at 6 and 12 months was 68.38% (95% CI 65.23–71.53) and 68.65% (95% CI 65.38–71.92), respectively, and for NARH was 90.73% (95% CI 88.74–92.72) and 87.76% (95% CI 85.45–90.07), respectively. The distribution of success for the two outcomes at 6 and 12 months according to the potential predictors among responders is reported in Table A1.

Table 2 shows the association measures between the potential predictors and AA and NARH obtained from univariate models. Alcohol abstinence in the month before RAARUs’ admission and the number of treatment’s strategies were identified as covariates to be included in the multivariate models of both outcomes, while education level was included only in the model for AA and the dual diagnosis only in the NARH’s model.

Table 3 shows the association for AA obtained from multivariate model. The results of the model showed that the outcome’s probability did not change over time as well as the effect of the covariates. Patients affected by alcohol-related comorbidities had a reduced probability of AA (RR 0.90, 95% CI 0.81–0.99). Patients already abstaining in the month before RAARUs’ admission had an increased probability of maintaining “abstainer” status even after discharge (RR 1.20, 95% CI 1.08–1.33). Finally, subjects undergoing more than four treatment strategies had an increased probability of being abstainers (RR 1.19, 95% CI 1.01–1.40) compared to patients undergoing less than two treatment strategies.

Table 4 shows the association measures for NARH outcomes obtained from the multivariate model. Patients with dual diagnosis had a decreased probability of NARH compared with patients without dual diagnosis (RR 0.95, 95% CI 0.91–0.99). A significantly increased probability of the outcome was observed among abstainers in the month before RAARUs’ admission compared to non-abstainers for the outcome measured at 12 months (RR 1.10; 95% CI 1.04–1.16), but this was not the case at six months. Finally, the number of treatment strategies did not affect the outcome at six months, while a significant reduction of the of the proportion of patients with no alcohol related hospitalizations (12%) was observed at 12 months for patients undergoing three or four treatment strategies. Even for NARH, the outcome’s probability did not change over time.

## 4. Discussion

The first aim of this study was to estimate the frequency of success (AA and NARH) at both six- and 12-month follow-up after alcohol residential rehabilitation programs in a large sample of alcohol dependent patients. In this study, we found that 68.38% and 68.65% of patients were abstinent at the six- and 12-month follow-up, respectively. Previous studies showed that about six out of ten patients with alcohol dependence will relapse in the six months following detoxification [14]. These relapse rates are certainly lower compared to those reported in the literature. This high rate of abstinence seems to be confirmed at 12-month follow-up and may suggest that this rehabilitation approach, characterized by short duration (28-day), high intensity and complexity of interventions (physical, psychological and family-related), multi-professional intervention (medical, psychological and educational services), and elaboration of an aftercare project [5,11,27] could have contributed to provide positive outcomes.

Additionally, this study explored the success of patients based on “no alcohol related hospitalization” and we found that the rate of success was 90.73% at six month follow-up and 87.76% at 12-month follow-up. To the best of our knowledge, few studies have explored the hospitalization of subjects diagnosed with alcohol dependence. For example, a recent study [31] looked at a large cohort of Italian alcoholics over a five-year period and found high rates of hospitalizations mainly for mental disorders, diseases of the digestive system, accidents or violence; this study confirmed the association of alcohol dependence with several medical conditions and its impact on health and social costs. The low rates of hospitalizations in this study can be explained by the high rate of abstainers in this sample and by the relatively short-term period of follow-up. For example, a previous study showed that after three months of treatment, most patients were abstinent and only 5% of the total sample were hospitalized for alcohol dependence [32]. Miquel et al. [33] also examined the association between drinking levels and inpatient health service utilization in individuals with a lifetime diagnosis of alcohol dependence, and found that admission rates were lowest for abstainers compared to people with moderate and heavy drinking. These findings suggest that alcohol treatment can reduce overall medical costs of alcohol dependence, with particular regard for patients under 50 years of age and when the treatment is effective [32,33,34,35].

The second aim was to identify the predictors of success of alcohol rehabilitation programs by examining various sociodemographic and clinical characteristics of patients, as well as the number of treatment strategies they received. When we look at predictors of abstinence, results of this study showed that having alcohol-related comorbidities reduced the probability of being abstainers after discharge from RAARUs. This result can be explained by the fact that this variable can be a proxy of the severity of alcohol dependence or abuse and, therefore, patients with a more severe addiction may be more prone to relapses than subjects with less severe addiction problems. Moreover, being abstainers in the month before the rehabilitation and undergoing more than for treatment strategies (compared to less than two) increased the probability of success both at six and 12 months. It can be supposed that abstinent patients in the last month before recovery had higher levels of motivation and readiness to change their drinking behaviours compared to other patients. Previous studies showed that an individual’s readiness to change and motivation for treatment have a key role in predicting attendance, recovery, and prognosis [4,36,37]. Finally, findings of this study showed that an increasing number of treatment strategies, representing both the multidisciplinarity and intensity of the treatment, had a significant effect on patients’ abstinence at follow-up. During the last 20 years in Italy there had been an increased presence of short residential treatment facilities (1–6 months) for the treatment of alcohol addiction [28]. The characteristics of the residential treatment were medical, pharmacological, educational and psychotherapeutic interventions characterized by short duration and complex intense therapeutic intervention strategies, mainly addressed to patients with severe clinical conditions, such as dual diagnosis [5]. No effect on abstinence was found for socio-demographic factors, such as age, education or occupation. Demographic factors were probably the most investigated in this research area, but mixed results were obtained and results from a review [16] suggested that the most consistent predictors of alcohol-related outcomes did not involve socio-demographic characteristics.

With regard to predictors of no alcohol related hospitalization, results showed a significant positive effect of abstinence in the month before RAARU’s admission. Additionally, findings showed that patients with dual diagnosis had a decreased probability of no alcohol related hospitalization compared to other patients. The increased risk of patients with dual diagnosis for alcohol related hospitalization can be explained by the fact that they are more likely to seek treatment than individuals with a single disorder because of the functional impairment and distress they experience from their co-occurring disorder [27]. It is well known that treatment of patients with dual diagnosis is more challenging: They are more likely to have significant deterioration in social functioning, suffer from medical disorders, fail to maintain a stable financial situation, and come into conflict with the law [30]. In consideration of the increased number of alcohol dependent patients with dual diagnosis, it is important to develop “share care” models—integrated treatments which pay attention to the complexity of the patients—and aim to plan an aftercare project in order to keep them engaged in the care path.

An unexpected decreased probability of NARH at 12 months was found for patients receiving three to four treatment strategies. The interpretation of this result is very difficult and indicates the need of future studies exploring the specific role of treatment strategies on patients’ prognosis; we could suppose that patients who underwent intense treatment learn the importance of seeking medical help and treatments, rather than self-medicating, which is very common in the context of substance abuse.

Some limitations of our study should be mentioned. First, the proportion of subjects (20–25%) who declined to be interviewed at follow-up may have introduced a bias in our results. However, weighting the models for the inverse probability of being a non-responder should have reduced the impact of non-response on the association estimates. Secondly, outcomes of this study (abstinence and no alcohol related hospitalization) were assessed by a trained health worker using only a phone interview; therefore, we cannot exclude the possibility that the estimated association measures are affected by misclassification of the outcome. However, phone interviews have already been used by several previous studies [8,30,38]. Thirdly, we have included in our analyses some socio-demographic and clinical factors, but we cannot exclude the possibility that other variables not measured in this study could be associated with our outcomes. Finally, we hypothesized that the contribution of the specific rehabilitation model explains our results, but we would need a control group in order to explore the effectiveness of the rehabilitative approach itself.

## 5. Conclusions

The results of this study are encouraging because they show that a significant proportion of alcohol dependent patients were abstinent and did not require hospital and/or emergency room admission for alcohol related problems at both six- and 12-month follow-ups after residential rehabilitation. We found that many alcohol dependent patients had a dual diagnosis and this confirmed the increment of patients admitted to rehabilitation programs for alcohol dependence with psychiatric diagnoses and/or with polysubstance abuse [34]. This suggests that we should pay specific attention to the type of treatment with particular regard to a multidisciplinary approach in order to respond to the complexity of these patients. Specifically, this study found that being abstinent the month before rehabilitation and the intensity of the treatment, which combined medical, psychological, educational and physical interventions has a significant positive effect on abstinence at follow-up. Future research is needed in order to explore the effectiveness of the different potential treatment strategies on abstinence rates. This would allow us to identify specific groups of patients achieving poorer outcomes in order to improve accuracy of prognosis and design tailored interventions based on patients’ needs.

## Figures and Tables

**Table 1:** Distribution of responders and association with several socio demographic and clinical characteristics and the number of treatment’s strategies at 6 and 12 months for alcohol abstinence and no alcohol related hospitalizations.

Variables	Alcohol Abstinence				No Alcohol Related Hospitalizations °			
	6		12		6		12	
	Months		Months		Months		Months	
	Non Responders *N* = 205	Responders *N* = 835	Non Responders *N* = 265	Responders *N* = 775	Non Responders *N* = 220	Responders *N* = 820	Non Responders *N* = 264	Responders *N* = 776
	*N* (%)	*N* (%)	*N* (%)	*N* (%)	*N* (%)	*N* (%)	*N* (%)	*N* (%)
Socio demographic characteristic								
Age								
≤40 years	84 (40.98)	249 (29.82)	104 (39.25)	229 (29.55)	85 (38.64)	248 (30.24)	105 (39.77)	228 (29.38)
41–50 years	75 (36.59)	286 (34.25)	93 (35.09)	268 (34.58)	79 (35.91)	282 (34.39)	93 (35.23)	268 (30.38)
>50 years	46 (22.44)	300 (35.93)	68 (25.66)	278 (35.87)	56 (25.45)	290 (35.37)	66 (25)	280 (31.38)
*p*-value	0.0004		0.0025		0.0108		0.0009	
Gender								
Male	155 (75.61)	590 (70.66)	193 (73.11)	552 (71.13)	166 (75.45)	579 (70.61)	193 (73.11)	552 (71.13)
Female	50 (24.39)	245 (29.34)	71 (26.79)	224 (28.9)	54 (24.55)	241 (29.39)	71 (26.89)	224 (28.87)
*p*-value	0.1588		0.5105		0.1569		0.5392	
Education ^§^								
Low	27 (13.17)	136 (16.29)	32 (12.08)	131 (16.9)	33 (15)	130 (15.85)	31 (11.74)	132 (17.01)
Medium	103 (50.24)	403 (48.26)	126 (47.55)	380 (49.03)	111 (50.45)	395 (48.17)	125 (47.35)	381 (49.1)
High	75 (36.59)	296 (35.45)	107 (40.38)	264 (34.06)	76 (34.55)	295 (35.98)	108 (40.91)	263 (33.89)
*p*-value	0.5454		0.0731		0.8327		0.0422	
Occupation								
Unemployed	83 (40.49)	290 (34.73)	105 (39.62)	268 (34.58)	90 (40.91)	283 (34.51)	105 (39.77)	268 (34.54)
Employed	105 (51.22)	407 (48.74)	127 (47.92)	385 (49.68)	108 (49.09)	404 (49.27)	127 (48.11)	385 (49.61)
Retired	17 (8.29)	138 (16.53)	33 (12.45)	122 (15.74)	22 (10)	133 (16.22)	32 (12.12)	123 (15.85)
*p*-value	0.01		0.228		0.0391		0.1795	
Clinical characteristics								
Comorbidities *								
No	126 (61.46)	476 (57.01)	131 (49.43)	471 (60.77)	160 (72.73)	442 (53.9)	159 (60.23)	443 (57.09)
Yes	79 (38.54)	359 (42.99)	89 (33.58)	349 (45.03)	105 (47.73)	333 (40.61)	105 (39.77)	333 (42.91)
*p*-value	0.2468		0.5742		0.3411		0.3721	
Alcohol abstinence in the last month before recovery								
No	37 (18.05)	181 (21.68)	41 (11.23)	177 (22.84)	176 (80)	646 (78.78)	224 (84.85)	598 (77.06)
Yes	168 (81.95)	654 (78.32)	224 (61.37)	598 (77.16)	44 (20)	174 (21.22)	40 (15.15)	178 (22.94)
*p*-value	0.2528		0.011		0.6931		0.0073	
Polysubstance abuse								
No	141 (68.78)	642 (76.89)	184 (50.41)	599 (77.29)	154 (70)	629 (76.71)	183 (69.32)	600 (77.32)
Yes	64 (31.22)	193 (23.11)	81 (22.19)	176 (22.71)	66 (30)	191 (23.29)	81 (30.68)	176 (22.68)
*p*-value	0.0159		0.0105		0.0406		0.0092	
Dual diagnosis ^&^								
No	108 (52.68)	491 (58.8)	140 (38.36)	459 (59.23)	119 (54.09)	480 (58.54)	138 (52.27)	461 (59.41)
Yes	97 (47.32)	344 (41.2)	125 (34.25)	316 (40.77)	101 (45.91)	340 (41.46)	126 (47.73)	315 (40.59)
*p*-value	0.1121		0.069		0.2361		0.0427	
Treatments								
Number of treatment’ strategies								
≤2	25 (12.2)	94 (11.26)	38 (10.41)	81 (10.45)	24 (10.91)	95 (11.59)	38 (14.39)	81 (10.44)
3–4	77 (37.56)	310 (37.13)	99 (27.12)	288 (37.16)	93 (42.27)	294 (35.85)	97 (36.74)	290 (37.37)
>4	103 (50.24)	431 (51.62)	128 (35.07)	406 (52.39)	103 (46.82)	431 (52.56)	129 (48.86)	405 (52.19)
*p*-value	0.907		0.1966		0.2118		0.2081	

° Access to emergency room (ER) or hospital admission for alcohol related causes. ^§^ Low: No education or primary school degree; medium: Secondary school degree; high: High school degree or bachelor’s degree. ^&^ Presence of at least a diagnosis among drugs dependence or abuse, personality disorders, affective syndrome, anxious and reactive syndrome, schizophrenic psychosis, impulse control disorder, dementia and oligophrenia in addiction to alcohol abuse. * Patients with at least a diagnosis among cirrhosis, liver disease except cirrhosis, polyneuropathy and other comorbidities.

**Table 2:** Associations between sociodemographic characteristics, clinical characteristics, and the number of treatment strategies and alcohol abstinence and no alcohol related hospitalization at six and 12 months, using a univariate model.

Variable	Alcohol Abstinence		No Alcohol Related Hospitalizations °	
	RR (95% CI)	*p*-Value	RR (95% CI)	*p*-Value
Socio demographic characteristic				
Education ^§^				
Low	1.00		1.00	
Medium	0.89 (0.79–1.00)	0.0578	0.99 (0.94–1.03)	0.3408
High	0.87 (0.76–1.00)	0.0476	0.97 (0.92–1.03)	0.5381
Occupation				
Unemployed	1.00		1.00	
Employed	1.05 (0.94–1.17)	0.3945	1.00 (0.96–1.04)	0.8785
Retired	1.06 (0.92–1.23)	0.4164	1.00 (0.95–1.06)	0.9219
Clinical characteristics				
Comorbidities *				
No	1.00		1.00	
Yes	0.93 (0.84–1.03)	0.184	0.99 (0.96–1.03)	0.7353
Alcohol abstinence in the last month before recovery				
No	1.00		1.00	
Yes	1.18 (1.07–1.31)	0.0009	1.05 (1.01–1.09)	0.0054
Polysubstance abuse				
No	1.00		1.00	
Yes	1.02 (0.90–1.14)	0.7781	1.01 (0.97–1.05)	0.6659
Dual diagnosis ^&^				
No	1.00		1.00	
Yes	0.97 (0.87–1.07)	0.5034	0.95 (0.91–0.99)	0.019
Number of treatment strategies				
≤2	1.00		1.00	
3–4	0.85 (0.72–1.00)	0.0507	1.00 (0.95–1.06)	0.9881
>4	0.96 (0.86–1.07)	0.4739	0.96 (0.92–1.00)	0.0488

^§^ Low: No education or primary school degree; medium: Secondary school degree; high: High school degree or bachelor’s degree. * Patients with at least a diagnosis among cirrhosis, liver disease except cirrhosis, polyneuropathy and other comorbidities. ^&^ Presence of at least a diagnosis among drugs dependence or abuse, personality disorders, affective syndrome, anxious and reactive syndrome, schizophrenic psychosis, impulse control disorder, dementia and oligophrenia in addiction to alcohol abuse. ° Access to ER or hospital admission for alcohol related causes. RR: relative risk, 95% CI: 95% confidence interval. Categorical variables with with a *p*-value ≤ 0.2 are eligible for inclusion in the multivariate model.

**Table 3:** Association between sociodemographic and clinical characteristics, and the number of treatment strategies and alcohol abstinence at six and 12 months, using multivariate models.

Variable	Alcohol Abstinence	
	RR (95% CI)	*p*-Value
Socio demographic characteristic		
Age		
≤40 years	1.00	
41–50 years	0.94 (0.83–1.07)	0.3530
>50 years	1.07 (0.94–1.21)	0.3029
Gender		
Female	1.00	
Male	1.08 (0.97–1.21)	0.1643
Education ^§^		
Low	1.00	
Medium	0.93 (0.82–1.05)	0.2262
High	0.91 (0. 78–1.05)	0.1740
Clinical characteristics		
Comorbidities *		
No	1.00	
Yes	0.90 (0.81–0.99)	0.0319
Alcohol abstinence in the last month before recovery		
No	1.00	
Yes	1.20 (1.08–1.33)	0.0009
Number of treatment’s strategies		
≤2	1.00	
3–4	1.08 (0.92–1.28)	0.3450
>4	1.19 (1.01–1.40)	0.0376
Time		
6 months	1.00	
12 months	0.97 (0.93–1.01)	0.1175

^§^ Low: No education or primary school degree; medium: Secondary school degree; high: High school degree or bachelor’s degree. * Patients with at least a diagnosis among cirrhosis, liver disease except cirrhosis, polyneuropathy and other comorbidities. RR: relative risk; 95% CI: 95% confidence interval.

**Table 4:** Association between sociodemographic and clinical characteristics, and the number of treatment strategies and no alcohol related hospitalization at 6 and 12 months, using multivariate models.

Variable	No Alcohol Related Hospitalizations °	
	RR (95% CI)	*p*-Value
Socio demographic characteristic		
Age		
≤40 years	1.00	
41–50 years	0.99 (0.94–1.03)	0.5102
>50 years	1.01 (0.97–1.06)	0.6263
Gender		
Female	1.00	
Male	1.04 (1.00–1.09)	0.0556
Clinical characteristics		
Alcohol abstinence in the last month before recovery		
6 months		
No	1.00	
Yes	1.03 (0.99–1.07)	0.2107
12 months		
No	1.00	
Yes	1.10 (1.04–1.16)	0.0006
Dual diagnosis ^&^		
No	1.00	
Yes	0.95 (0.91–0.99)	0.0253
Number of treatment’s strategies		
6 months		
≤2	1.00	
3–4	1.00 (0.93–1.07)	0.9591
>4	1.01 (0.94–1.08)	0.7852
12 months		
≤2	1.00	
3–4	0.88 (0.82–0.96)	0.0015
>4	0.97 (0.94–1.00)	0.0536
Time		
6 months	1.00	
12 months	1.01 (0.94–1.09)	0.7922

^&^ Presence of at least a diagnosis among drugs dependence or abuse, personality disorders, affective syndrome, anxious and reactive syndrome, schizophrenic psychosis, impulse control disorder, dementia and oligophrenia in addiction to alcohol abuse. ° Access to ER or hospital admission for alcohol related causes. RR: relative risk, 95%CI: 95% confidence interval.

## Appendix A

**Table A1:** Proportion of outcomes among responders according to several sociodemographic and clinical characteristics and the number of treatment strategies at 6 and 12 months.

Characteristics	Abstinence Among Reponders		No Alcohol Related Hospitalization ° Among Reponders	
	6 Months *N* = 835	12 Months *N* = 820	6 Months *N* = 775	12 Months *N* = 776
	Success *N* (%)	Success *N* (%)	Success *N* (%)	Success *N* (%)
Socio demographic characteristics				
Age				
≤40 years	162 (28.37)	166 (31.20)	223 (29.97)	202 (29.66)
41–50 years	186 (32.57)	170 (31.95)	253 (34.01)	229 (33.63)
>50 years	223 (39.05)	196 (36.84)	268 (36.02)	250 (36.71)
Gender				
Male	411 (71.98)	384 (72.18)	534 (71.77)	491 (72.1)
Female	160 (28.02)	148 (27.82)	210 (28.23)	190 (27.9)
Education				
Low	100 (17.51)	93 (17.48)	122 (16.4)	117 (17.18)
Medium	272 (47.64)	256 (48.12)	360 (48.39)	335 (49.19)
High	199 (34.85)	183 (34.40)	262 (35.22)	229 (33.63)
Occupation				
Unemployed	191 (33.45)	187 (35.15)	254 (34.14)	232 (34.07)
Employed	282 (49.39)	265 (49.81)	367 (49.33)	342 (50.22)
Retired	98 (17.16)	80 (15.04)	123 (16.53)	107 (15.71)
Clinical characteristics				
Comorbidities *				
No	329 (57.62)	315 (59.21)	431 (57.93)	392 (57.56)
Yes	242 (42.38)	217 (40.79)	313 (42.07)	289 (42.44)
Alcohol abstinence in the last month before recovery				
No	432 (75.66)	399 (75.00)	584 (78.49)	515 (75.62)
Yes	139 (24.34)	133 (25.00)	160 (21.51)	166 (24.38)
Polysubstance abuse				
No	436 (76.36)	407 (76.50)	571 (76.75)	525 (77.09)
Yes	135 (23.64)	125 (23.50)	173 (23.25)	156 (22.91)
Dual diagnosis				
No	342 (59.89)	321 (60.34)	450 (60.48)	414 (60.79)
Yes	229 (40.11)	211 (39.66)	294 (39.52)	267 (39.21)
Number of treatments’ strategies				
≤2	53 (9.28)	53 (9.96)	86 (11.56)	76 (11.16)
3–4	209 (36.60)	183 (34.40)	266 (35.75)	246 (36.12)
>4	309 (54.12)	296 (55.64)	392 (52.69)	359 (52.72)

* Patients with at least a diagnosis among cirrhosis, liver disease except cirrhosis, polyneuropathy and other comorbidities. ° Access to ER or hospital admission for alcohol related causes.
